# Catatonia induced by abrupt discontinuation of clozapine - case report

**DOI:** 10.1192/j.eurpsy.2021.1452

**Published:** 2021-08-13

**Authors:** C. Oliveira, F. Caldas, M. Gonçalves

**Affiliations:** Internamento C, Hospital de Magalhães Lemos, Porto, Portugal

**Keywords:** abrupt interruption of clozapine, rebound effects, Catatonia, clozapine

## Abstract

**Introduction:**

Catatonia is characterized by a bizarre and severe psychomotor change. According to DSM-5, the presence of three or more symptoms is necessary to affirm the diagnosis: stupor, catalepsy, brain flexibility, mutism, negativism, posturing, mannerisms, stereotypes, agitation not influenced by external stimuli, grimaces, echolalia or echopraxia. The association between first- and second-generation antipsychotics (AP) and the onset of catatonia is well established in the literature. In contrast, clozapine is one of the second-generation APs that is recognized for its effectiveness in treating catatonia, rather than inducing it. However, it has been documented that abrupt discontinuation of clozapine can induce rapid clinical deterioration with multiple presentations including: psychoses, cholinergic rebound states, serotonergic syndromes and catatonia.

**Objectives:**

Review the literature on catatonia associated with abrupt interruption of clozapine. Describe a clinical case.

**Methods:**

Observation of the patient and consultation the clinical file. Non-systematic literature review on catatonia, clozapine, side effects associated with rapid discontinuation and respective treatment.

**Results:**

34-year-old man, with the diagnosis of Schizoaffective Disorder. Admitted due to an acute decompensation with psychotic symptoms resistant to treatment requiring the introduction of clozapine. In the absence of a clinical response, clozapine was suspended, with the consequent appearance of catatonia resistant to benzodiazepines in high doses.

**Conclusions:**

Its already well established that the abrupt discontinuation of clozapine can trigger catatonia. This clinical case and literature review suits to emphasize the importance of educating psychiatrists on the adverse effects of psychiatric drugs and, in this case, the cautious discontinuation of clozapine in order to avoid its rebound effects.

